# Current status and trends of gut microbiota in the study of cognitive impairment in the elderly

**DOI:** 10.3389/fmicb.2026.1910406

**Published:** 2026-10-27

**Authors:** Ming-Yue Sun, Ying Wang, Jun Jiang, Shuo Feng, Yin-Gui Sun

**Affiliations:** 1 Department of Anesthesiology, Shandong Second Medical University, Weifang, Shandong, China; 2 Department of Anesthesiology, Affiliated Hospital of Shandong Second Medical University, Weifang, Shandong, China; 3 Affiliated Hospital of Shandong Second Medical University, Weifang, Shandong, China

**Keywords:** bibliometrics, brain-gut axis, cognitive impairment, gut microbiota, older adults

## Abstract

**Background:**

Cognitive impairment in older persons has become a major public health concern as the world’s population ages more quickly. A reduction in thinking, learning, and memory skills that affects a person’s day-to-day activities or social functioning is referred to as cognitive impairment. The connection between gut microbiota and cognitive decline has attracted a lot of interest lately. However, this rapidly expanding interdisciplinary field presents an increasingly complex knowledge structure, with existing evidence being highly fragmented and subject to significant methodological and sample size limitations.

**Goal:**

This study adopts a bibliometrics perspective, summarising and outlining the current state and developmental trends within the field to provide a reference framework and recommendations for subsequent research.

**Method:**

Based on the Web of Science Core Collection (WoSCC) database and Scopus database. In WOSCC database, literature related to gut microbiota and cognitive impairment in the elderly from 1995 to 8 September 2025 was retrieved. Similarly, search for relevant literature from 1995 to 2025 in the Scopus database. Bibliometric analysis was conducted using VOSviewer software to examine data on countries, institutions, authors, journals, keywords, citations, and generate visual maps.

**Result:**

A total of 406 relevant publications were retrieved from the WOSCC database. The overall publication trend is increasing. China is the country with the most published papers, and Nanjing Medical University and Zhejiang University are joint top institutions in terms of output, while in the WOS database, Hariom Yadav is the most prolific author, while Luo Ailin has the highest total link strength. In the Scopus database, Zhang, Y is the most prolific author, while Hisada, T et al. have the highest total link strength. The most common keyword is gut microbiota, followed by Alzheimer’s disease, aging, probiotics, dementia, and neuroinflammation. In addition, a total of 180 relevant literatures were retrieved from the Scopus database. Among them, the most common keywords are intestine flora, aged, cognitive defect, cognition, and gastrointestinal microbiome.

**Conclusion:**

Research into the relationship between the gut microbiota and cognitive impairment is advancing steadily, yet there remains a need to strengthen collaboration between authors and institutions across different regions. Sustained interaction and exchange are essential to further explore the mechanisms of action of the gut microbiota. Based on the identified research hotspots and gaps, we suggest that future investigations could benefit from integrating multi-omics approaches to explore potential biomarkers and nutritional intervention strategies. However, these directions remain speculative and should be pursued through rigorous experimental and clinical research.

## Introduction

With the acceleration of global aging, the number of elderly people is increasing. Since aging is the greatest risk factor for cognitive impairment in older adults (Thakur et al., 2025). Alzheimer’s disease, vascular dementia, mild cognitive impairment, and other conditions are common age-related cognitive diseases (Pérez Palmer et al., 2022). Additionally, more older people are undergoing anesthetic operations, which raises the risk of postoperative cognitive impairment. According to clinical data, one-third of older patients will have long-term functional impairment, and 10 to 60% will have postoperative cognitive dysfunction within a week following surgery (Lin et al., 2020; Liu C. L. et al., 2023). Oxidative stress, mitochondrial dysfunction, and neuroinflammatory factors are the causes of cognitive impairment (Sorrentino et al., 2017). The molecular pathologies include (1) oxidative stress-induced neuronal DNA damage, with abnormally elevated levels of the characteristic biomarker, 8-hydroxydeoxyguanosine (Liu et al., 2017); (2) reduced activity of mitochondrial complex IV, which triggers insufficient production of ATP (Kriebel et al., 2020); and (3) microglia overactivation triggers the release of TNF-α, IL-1β, and other pro-inflammatory factors (Jung et al., 2023).

The gut microbiota, which is one of the most abundant microbial communities in the human body, plays a crucial role in regulating metabolic, immune, and nervous system functions (Angelucci et al., 2019; Hsu et al., 2025). Imbalanced gut microbiota has been linked to cognitive impairment in the elderly (Nakai et al., 2021). The gut-brain axis refers to the enteric nervous system, also known as the “second brain,” which interacts with the central nervous system, autonomic nervous system, hypothalamic–pituitary–adrenal axis, and other structures to form a bidirectional regulatory pathway (Brunetti et al., 2019). This communication system closely links the central nervous system with the gut microbiota through multiple pathways, including neural, immune, endocrine, and metabolic mechanisms. Gut microbiota dysbiosis may contribute to neurodegenerative disorders (Bicknell et al., 2023). Potential pathways affecting cognitive function include (1) pro-inflammatory factors and reactive oxygen species (ROS) released by aberrant flora, inducing microglial cell activation and neuronal damage (Chen et al., 2021); (2) short-chain fatty acids (SCFAs), which regulate histone deacetylation and affect synaptic plasticity and expression of neurotrophic factors (Silva et al., 2020); (3) microbial toxins and metabolites produced by abnormal gut microbiota that act on the intestinal epithelium, leading to an increase in the permeability of the intestinal barrier, which then induces neuroinflammation and neurodegeneration in the central nervous system (Antushevich, 2020; Liang et al., 2018).

Bibliometric analysis of the field can visualize the development trend and dynamics of a certain field, reflecting the current research hotspots and cutting-edge directions (Chen and Song, 2019). Even while the number of studies on intestinal flora and cognitive impairment in older adults has grown significantly over the last 2 years, there are still few that use visualization analysis to identify hotspots and trends in this area. This study uses VOSviewer software to examine the pertinent literature and create visual maps based on the Web of Science Core Collection database and Scopus database with the goal of revealing the field’s development history, research hotspots, and future trends.

## Methodology

### Data sources

This study was based on Web of Science Core Collection, literature screening from Science Citation Index Expanded (SCI-EXPANDED)—1995–2025, Social Sciences Citation Index (SSCI)—1995–2025 two core databases, the search terms were TS = (“intestinal flora” OR “gut flora” OR “intestinal microflora” OR “gut microflora” OR “intestinal microbiota” OR “gut microbiota” OR “bowel microbiota” OR “bowel flora” OR “gut bacteria” OR “intestinal tract bacteria” OR “bowel bacteria” OR “gut metabolites” OR “gut microbiota”) AND TS = (“cognitive dysfunction” OR “cognitive impairment” OR “neurocognitive disorder” OR “cognitive decline” OR “mild cognitive impairment” OR “dementia” OR “vascular dementia”) AND TS = (“elderly” OR “elders” OR “elderly people” OR “aged” OR “older adults”). The search period was set from January 1995 to September 8, 2025, and the language was set to English. Initially, 413 articles were searched in the literature, and the article types were limited to Article and Review. By reading the titles and abstracts of the articles, 7 articles irrelevant to the research topic were deleted. After the above screening process, 406 documents were finally included, from which data such as country, institution, author, journal, keywords, and citation information were extracted for bibliometric analysis. As shown in Figure 1. Similarly, when searching for relevant literature from 1995 to 2025 in the Scopus database, there are a total of 180 articles.

**Figure 1:**
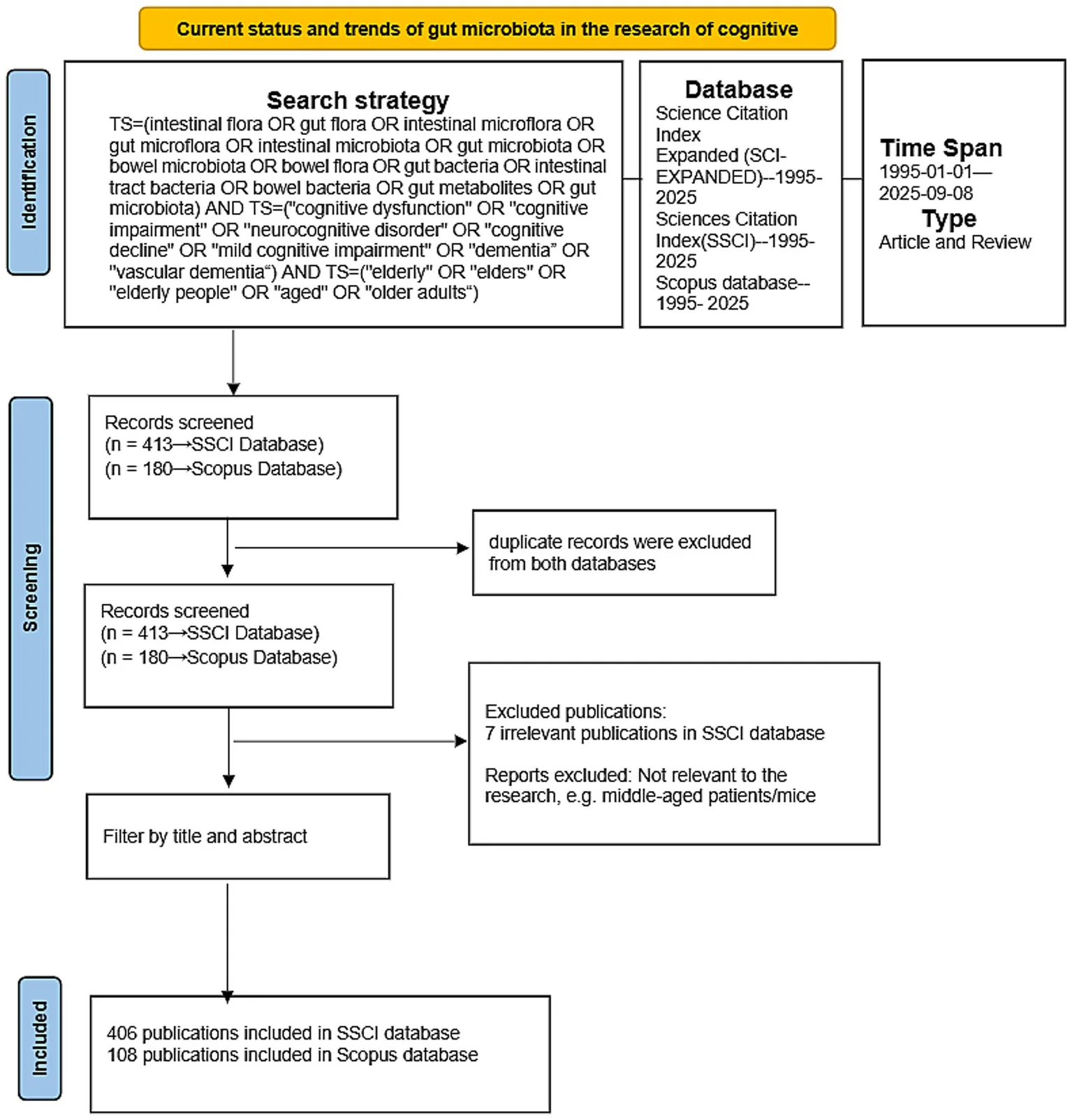
Data retrieval and analysis process.

### Inclusion and exclusion criteria

Inclusion criteria: (1) The research should also address the association between “gut microbiota” (such as gut microbiota, microbiota metabolites, etc.) and “cognitive impairment” (such as Alzheimer’s disease, mild cognitive impairment, etc.) and “old adults,” excluding literature that only discusses a single topic. Literature. For example, only select articles where the research subjects are “elderly,” manually excluding those where the subjects are “middle-aged” or “secondary school pupils”; (2) A time frame from January 1, 1995 to September 8, 2025, covering areas from the early stage to the latest research progress. (3) Types of included literature: original research articles and review articles. This study did not impose any restrictions on the type of study design; therefore, cohort studies, clinical trials, case–control studies, animal studies, and other types of studies were all included, provided their topics related to “gut microbiota,” “cognitive impairment,” and “the elderly.” Exclusion criteria: (1) Non-research literature (such as editorials, conference excerpts, news, letters to the editor, and other non-research or non-peer-reviewed documents. Etc.); (2) Duplicate publications or overlapping data; (3) Withdrawn literature; (4) Literature whose research content is irrelevant to the topic.

### Research tools

This study mainly used VOSviewer for bibliometric analysis. VOSviewer is a visual analysis tool launched by Van Eck and Waltman from Leiden University in the Netherlands in 2009 (van Eck and Waltman, 2017). Visual analysis of countries, institutions, authors, keywords, and co-citations of literature was conducted using VOSviewer (V1.6.19).

“VOSviewer Parameter Settings” Contents: 1. Clustering algorithm: VOS clustering technique. 2. Clustering resolution: resolution = 1.00. 3. Normalisation method: association strength normalisation. 4. Layout parameters: attraction = 2, repulsion = 0. 5. Number of random starts: random starts = 10. 6. Link counting method: full counting. 7. Handling of multi-author/multi-institution literature: collaborative literature is processed using fractional counting. 8. Exclusion of generic terms: a list of generic terms to be manually excluded is provided (e.g., ‘study’, ‘result’, ‘analysis’, etc.). The full-counting method may exaggerate the impact of teamwork on author rankings. Future studies could adopt the fractional-counting method or adjust the weighting of author contributions to partially mitigate this issue. The keyword source was selected as all keywords. The minimum number of publications for authors was set to 4, selecting the top ten individuals; the minimum publication threshold for institutions was set to 6; the minimum publication threshold for countries was set to 22. The minimum keyword occurrence count is 53. For the analysis unit, the analysis type for authors, institutions, and countries is set to co-authorship to examine collaborative relationships among authors, institutions, and countries, respectively. The analysis unit is all keywords, with the analysis type set to co-occurrence. Keywords have undergone synonym merging, e.g., ‘alzheimer’s disease’ replaces ‘alzheimers-disease’, and ‘gut microbiota’ replaces ‘gut microbiome’ and ‘intestinal microbiota’. The meanings of the various indicators are as follows: Publication count: the number of papers published by a country, organization or author; Citation count: the total number of times a paper has been cited; Total link strength: the total strength of the connections between a node and other nodes in a network; Centrality: the degree to which a node is central to the network structure.

## Results

### Number of annual publications

In the WOSCC database, a total of 406 pertinent papers were added after screening. The annual publishing volume and its shifting trend are depicted in Figure 2. There has been a notable three-stage evolution in this field: (1) The first core paper was published in 2009, and the yearly publishing volume was very low during the nascent period (2009–2016); (2) The development period (2017–2020) saw a progressive increase in the annual publication output, reaching over 31 articles in 2020. (3) Explosive period (2021–2025): Over 59 articles were written during this time, with an average of 64. Besides, in the Scopus database, a total of 180 pertinent papers were added after screening, the annual publishing volume and its shifting trend are depicted in Figure 3. The growth rate has dramatically increased since 2021, suggesting that the study of gut microbiota and cognitive impairment in the elderly has grown in importance during the last ten years.

**Figure 2:**
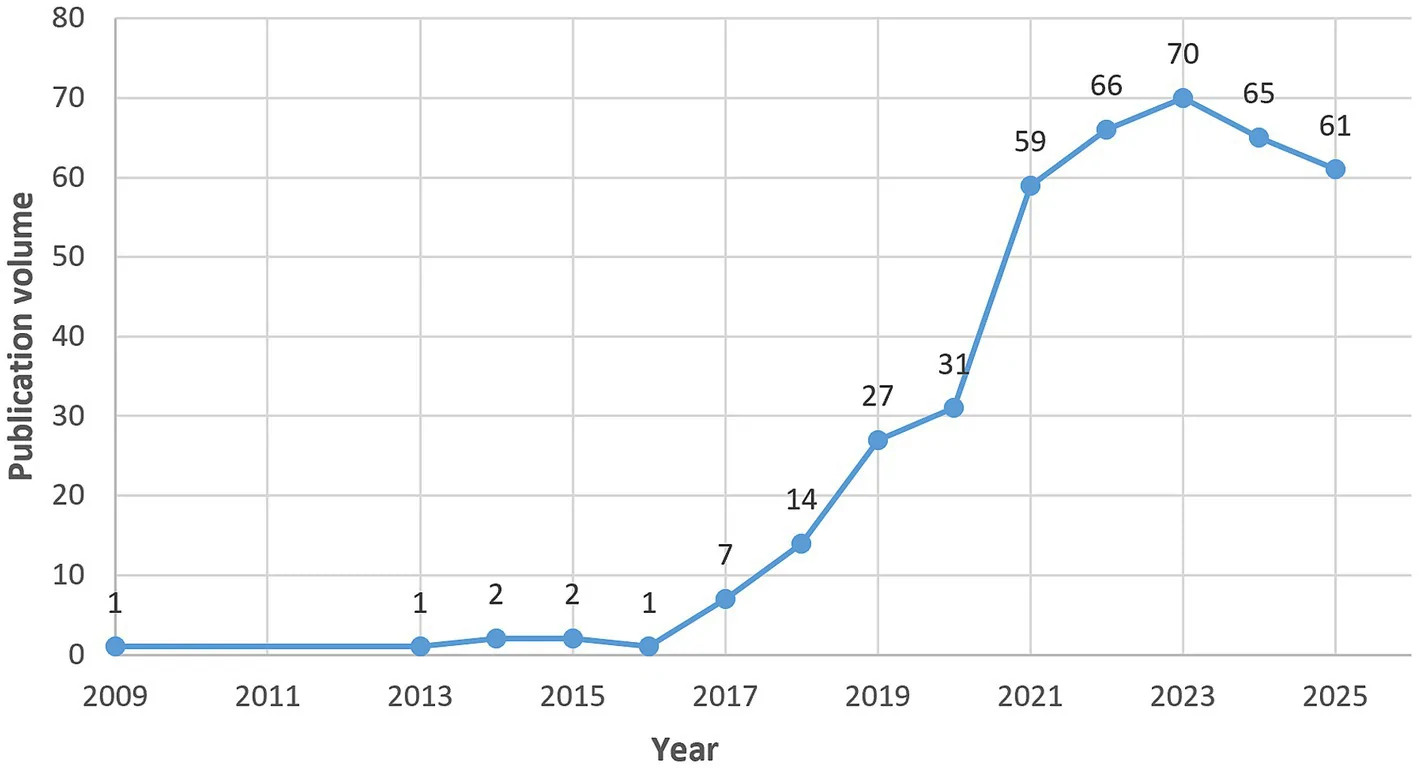
Annual publication trends in the Web of Science Core Collection Database (1995–2025). The numbers on the left represent the number of articles issued per year, and the numbers below represent the year.

**Figure 3:**
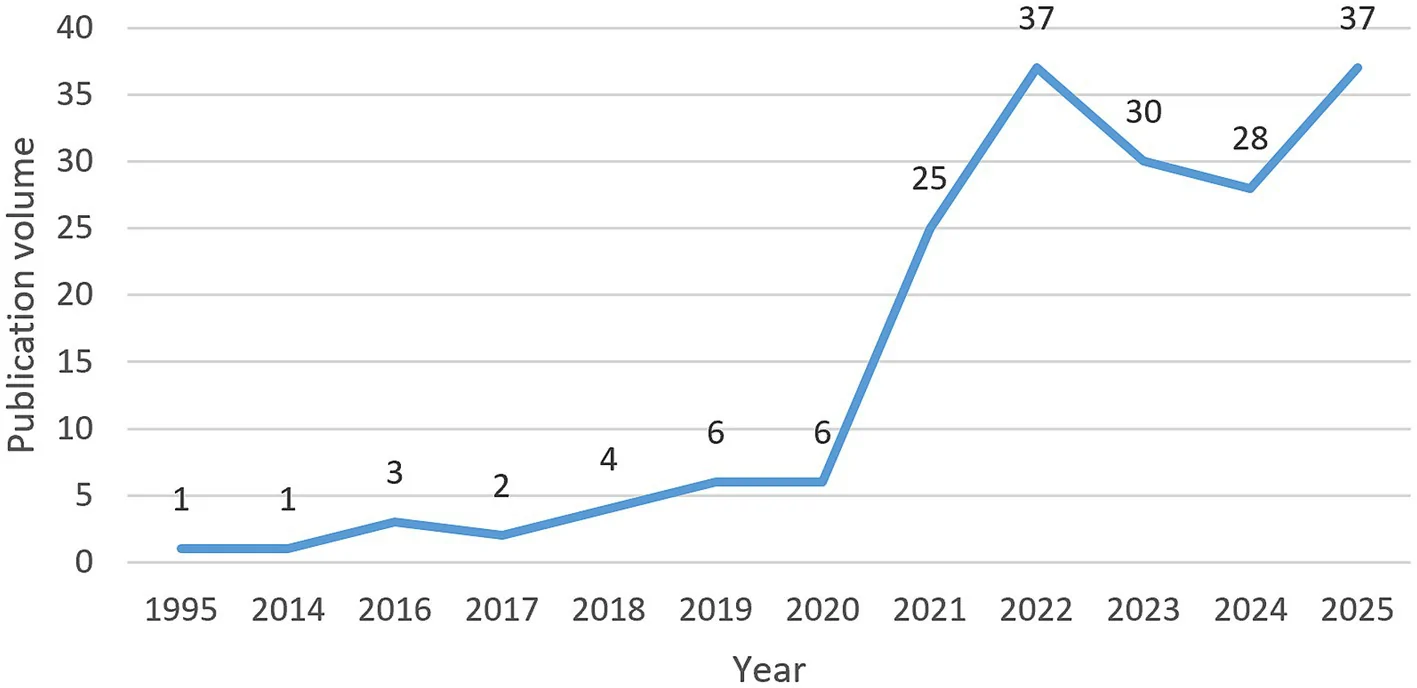
Annual publication trends in the Scopus database (1995–2025). The numbers on the left represent the number of articles issued per year, and the numbers below represent the year.

### Countries/regions

Table 1 lists the top five countries/regions in terms of the number of published articles and average citation in WOSCC database. Analysis shows that China ranks first with 186 papers, the United States second with 90, and the United Kingdom (29) and Italy (29) are tied for third place. In terms of average citations, the United Kingdom (70.14), Italy (69.00), and the United States (48.67) ranked the top three, with relatively high network centrality. Although China has laid the research foundation in this field with a large number of published papers, its average citation volume has not ranked among the top five. The United States performs well in both publication volume and average citation, demonstrating a balanced advantage in the scale of academic output and academic cooperation. The high average citation volume in the UK indicates its pivotal role in this discipline, effectively connecting research clusters in North America, Europe and the Asia-Pacific region, and playing a significant role in promoting cross-regional academic cooperation and theoretical dissemination. Figure 4 shows that the map of national/regional collaboration networks visualizes the global research cooperation landscape, indicating that there is a close collaboration network among countries/regions. Figure 5 shows that China has a relatively high research intensity in this field, with remarkable research investment and achievements.

**Table 1:** Top 5 countries/regions in terms of the number of publications in the WOSCC database.

Rank	Country	Documents	Citations	Average citation	Total link strength
1	Peoples R China	186	4,945	26.59	31
2	USA	90	4,380	48.67	40
3	England	29	2,034	70.14	25
4	Italy	29	2,001	69.00	16
5	South Korea	22	1,004	45.64	6

**Figure 4:**
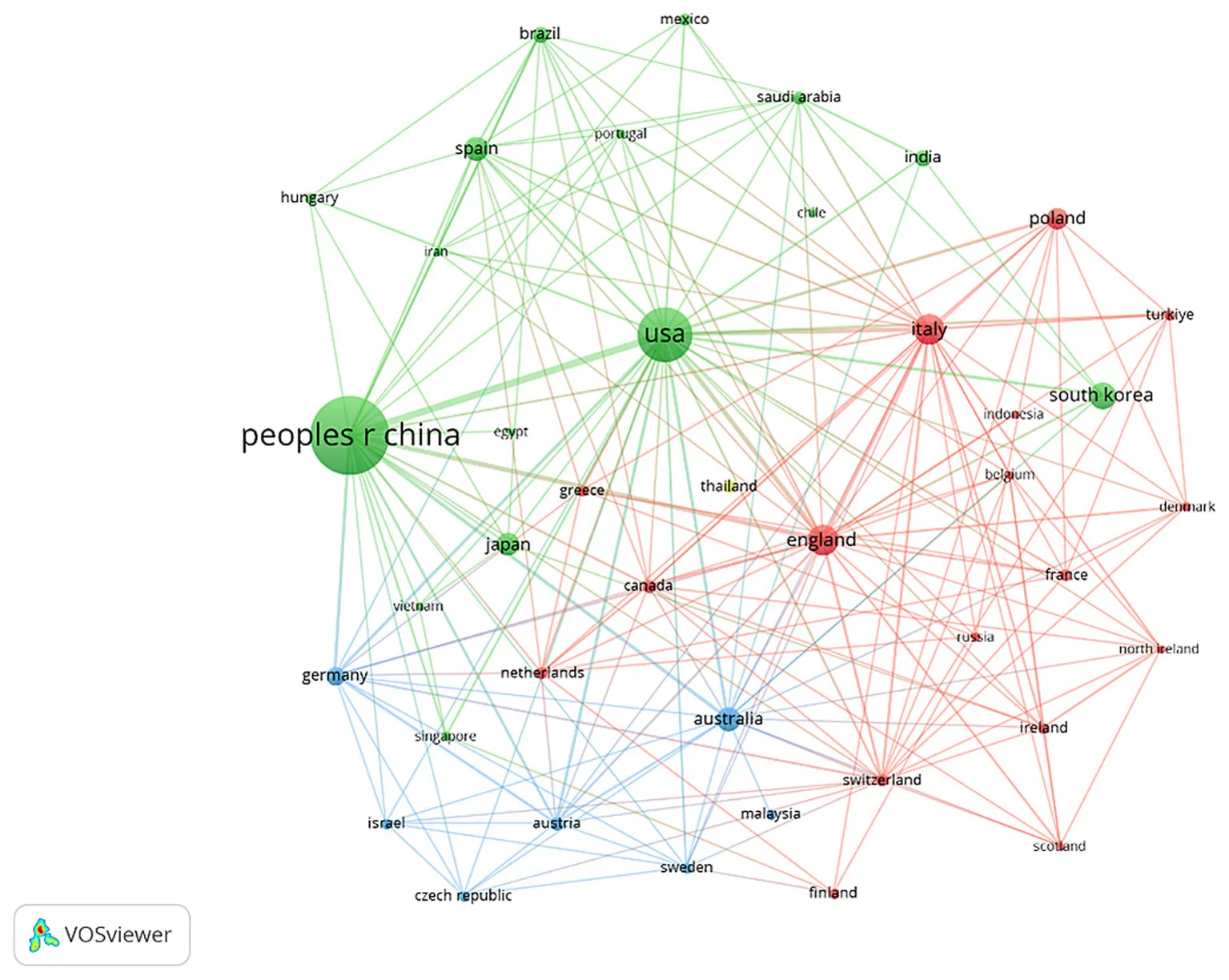
Cluster-based countries/regions collaboration map in the WOSCC database. The diameter of the circle is positively correlated with the number of national/regional publications, while the density of the connecting lines visualizes the intensity of national/regional cooperation.

**Figure 5:**
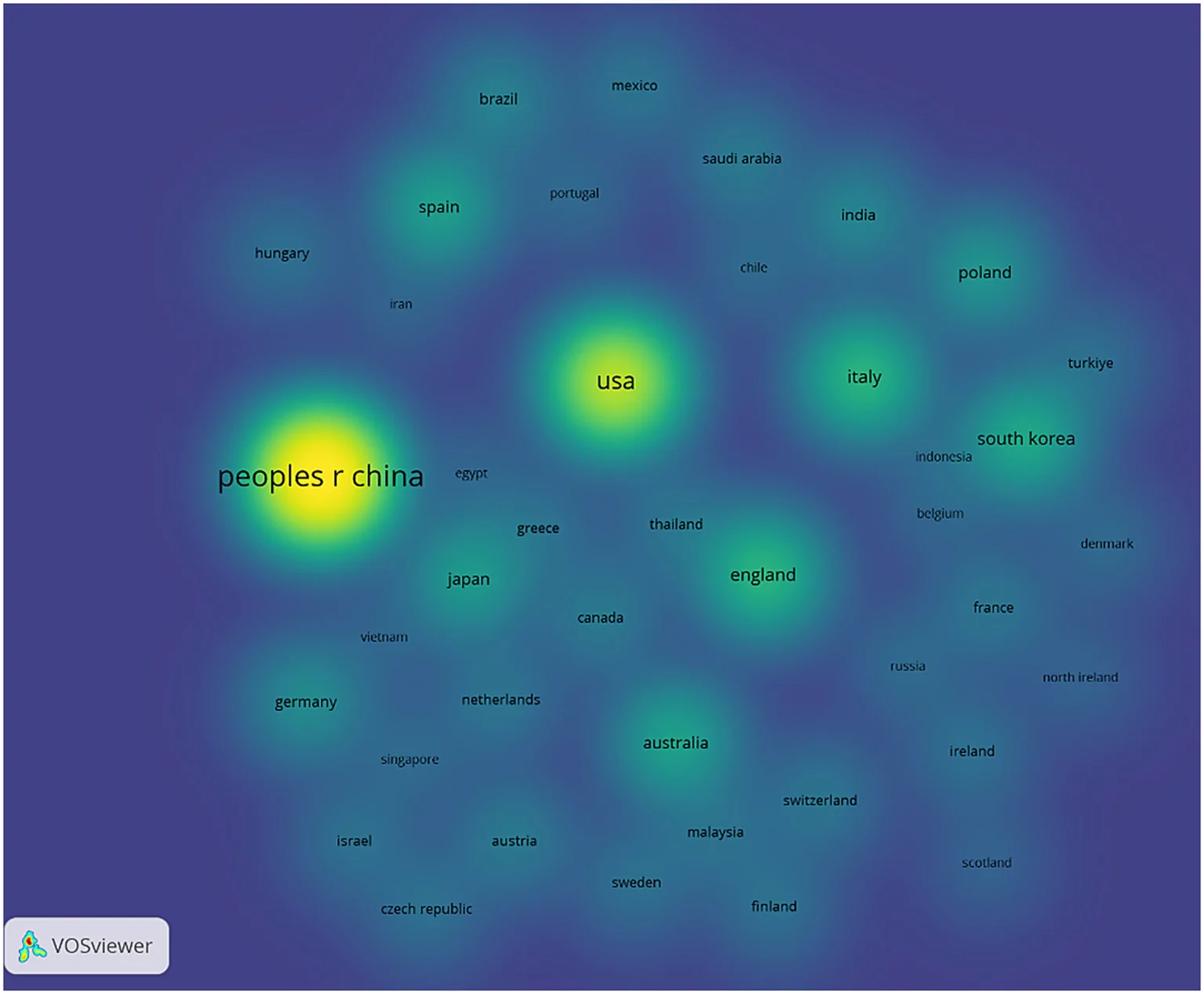
Countries/regions density map in the WOSCC database. The node size is related to the number of articles published in the country/region, and the node color is related to the number of articles published in recent years, the more the number of articles, the higher and the color brightness.

### Institutions

As can be seen from the co-occurrence network map of institutions in Figure 6 and the hotspot map in Figure 7, Nanjing Medical University and Zhejiang University are the institutions with the largest number of published papers and relatively high influence in this field, jointly publishing 11 papers. Huazhong university of Science and Technology and Capital Medical University followed closely behind, publishing 10 and 9 papers, respectively. Kyung hee university, Capital Medical University and Huazhong University of Science and Technology ranked the top three in the average citation ranking, with 49.00, 45.33 and 31.40, respectively. This indicates that the institution has relatively close exchanges and cooperation with other institutions. Table 2 presents the top ten institutions in terms of the number of articles and average citations. In the hotspot map of colleges and universities, Nanjing Medical University pays more attention to this field. Although Chinese institutions have an advantage in the number of papers (10 for Huazhong University of Science and Technology and 11 for Zhejiang University), their average institutional citation volume is relatively low. The high-output-low citation volume reflects the severe challenges that current research in the fields of gut microbiota and cognitive function in China is facing in the global knowledge network.

**Figure 6:**
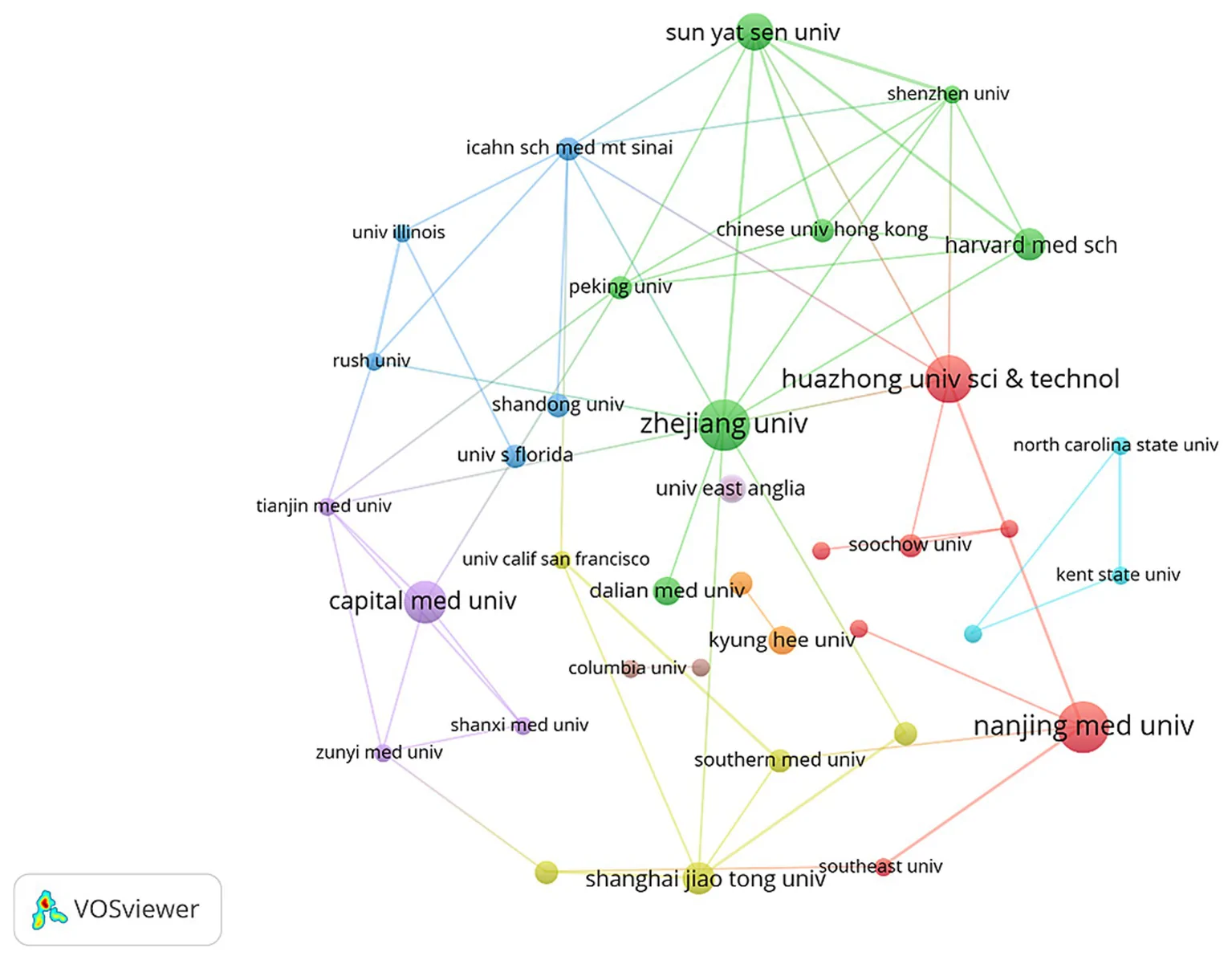
Cluster-based institutional collaboration map in the WOSCC database. The diameter of the circle is positively correlated with the number of papers issued by the institution, while the density of the connecting lines visualizes the intensity of institutional cooperation.

**Figure 7:**
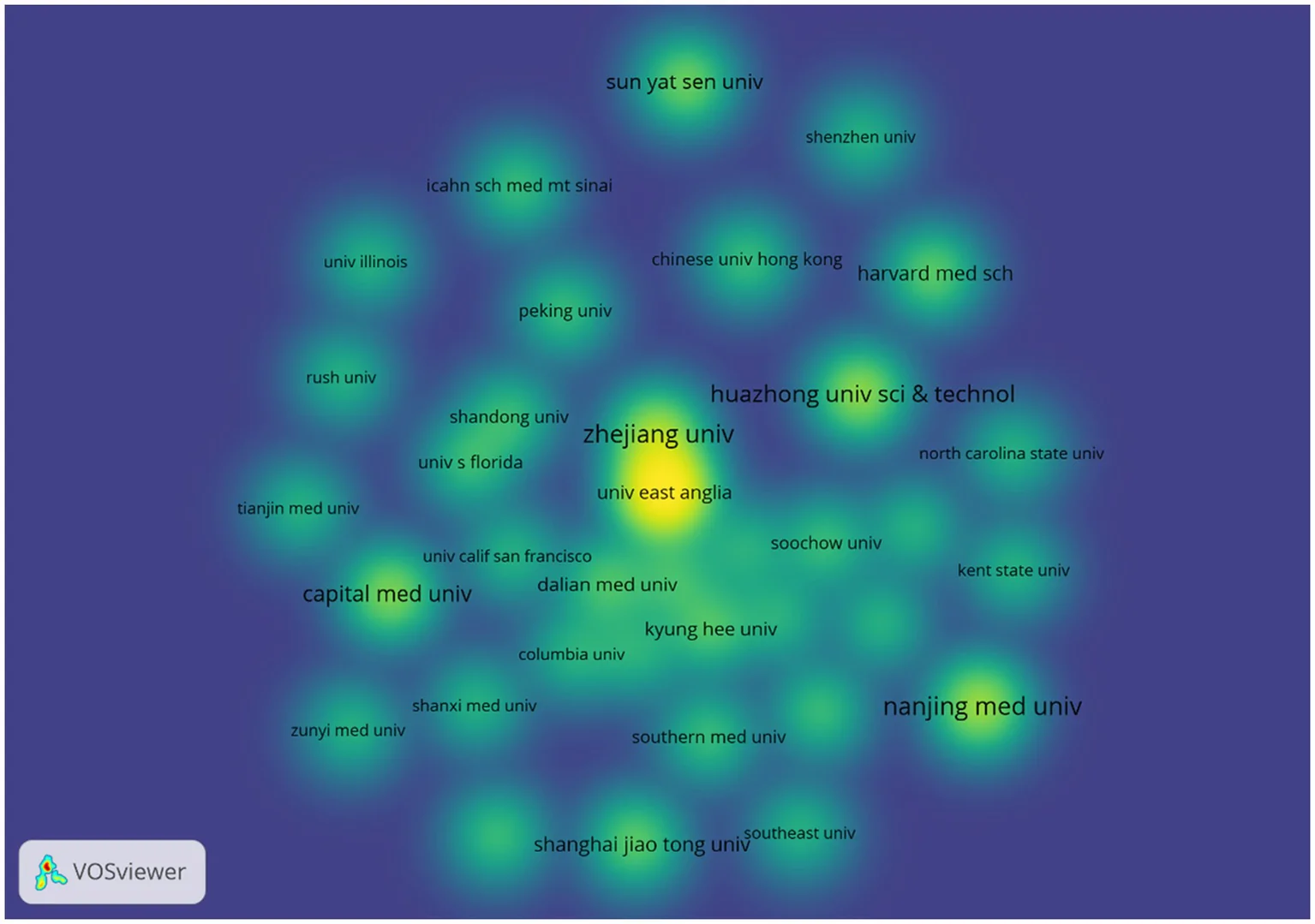
Institutional density map in the WOSCC database. The node size is related to the number of publications of the institution, and the node color is related to the number of publications in recent years, the more publications the higher the color brightness.

**Table 2:** Top 10 institutions in terms of the number of publications in the WOSCC database.

Rank	Organization	Documents	Citations	Average citation	Total link strength
1	Nanjing Med Univ	11	270	24.55	6
2	Zhejiang Univ	11	182	16.55	2
3	Huazhong Univ. Sci & Technol	10	314	31.40	4
4	Capital Med Univ	9	408	45.33	0
5	Sun Yat Sen Univ	8	221	27.63	5
6	Harvard Med Sch	7	203	29.00	3
7	Shanghai Jiao Tong Univ	7	122	17.43	1
8	Dalian Med Univ	6	109	18.17	1
9	Kyung Hee Univ	6	294	49.00	0
10	Univ East Anglia	6	56	9.33	0

### Authors

Table 3 presents the detailed information of the top 10 authors in terms of publication volume in the WOSCC database. The first one is yadav and hariom, who have published a total of 7 articles. The second and third places were, respectively, taken by luo, ailin (6 articles) and kim, dong-hyun (5 articles). hong, chang hyung, Dong Yuhua, jain, shalini, li, shiyong, nagpal, ravinder, vauzour, david and zhan, gaofeng tied for fourth place, each having published four articles. It demonstrates a continuous and stable ability to output scientific research. Figures 8, 9 show that the author-based research team has formed a close cooperative network. Through resource sharing and complementary advantages, scholars have formed close relationships, and teamwork has effectively promoted the rapid development of this field. Table 4 lists the top ten authors in terms of total link strength in the Scopus database. Among them, Zhang, Y is the author who has published the most articles, and Hisada, T is the author with the highest total link strength. In addition, Figure 10 shows the graph of author collaboration relationships. The scope of coverage differs between the two databases, resulting in discrepancies in the rankings of authors’ publication output. Total link strength reflects the level of activity within a collaborative network, the average number of citations reflects the average impact per paper, while the number of publications merely reflects the volume of output. Readers should consider multiple metrics together to understand an author’s performance.

**Table 3:** Top 10 authors in terms of the number of publications in the WOSCC database.

Rank	Author	Documents	Citations	Average citation	Total link strength
1	Yadav, Hariom	7	645	92.14	7
2	Luo, Ailin	6	220	36.67	11
3	Kim, Dong-Hyun	5	289	57.80	0
4	Hong, Chang Hyung	4	6	1.50	8
5	Hua, Dongyu	4	108	27.00	8
6	Jain, Shalini	4	31	7.75	7
7	Li, Shiyong	4	152	38.00	4
8	Nagpal, Ravinder	4	622	155.50	3
9	Vauzour, David	4	46	11.50	0
10	Zhan, Gaofeng	4	121	30.25	0

**Figure 8:**
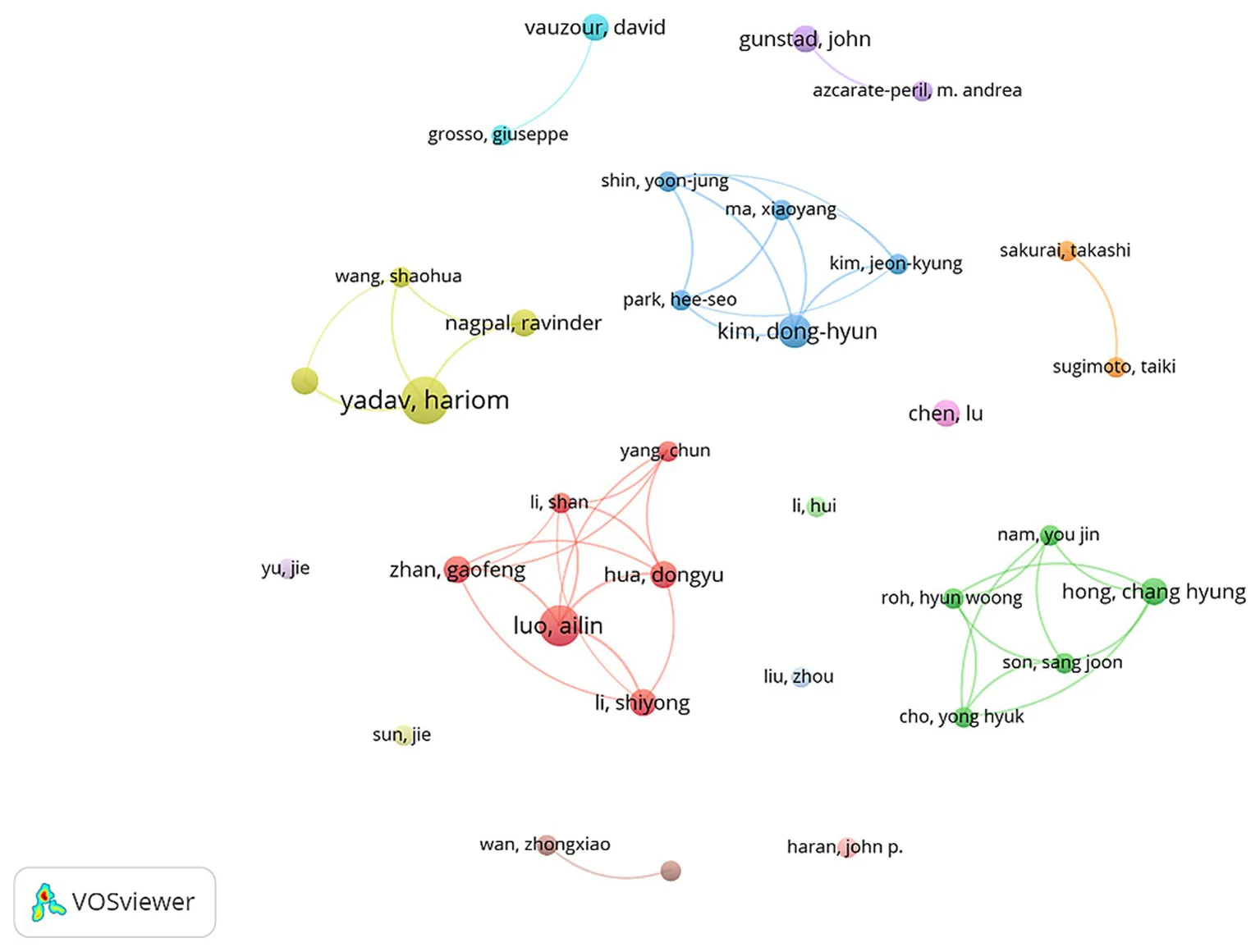
Cluster-based author collaboration map in the WOSCC database. The diameter of the circle is positively correlated with the number of publications by the authors, while the density of the connecting lines intuitively reflects the intensity of the collaboration between the authors.

**Figure 9:**
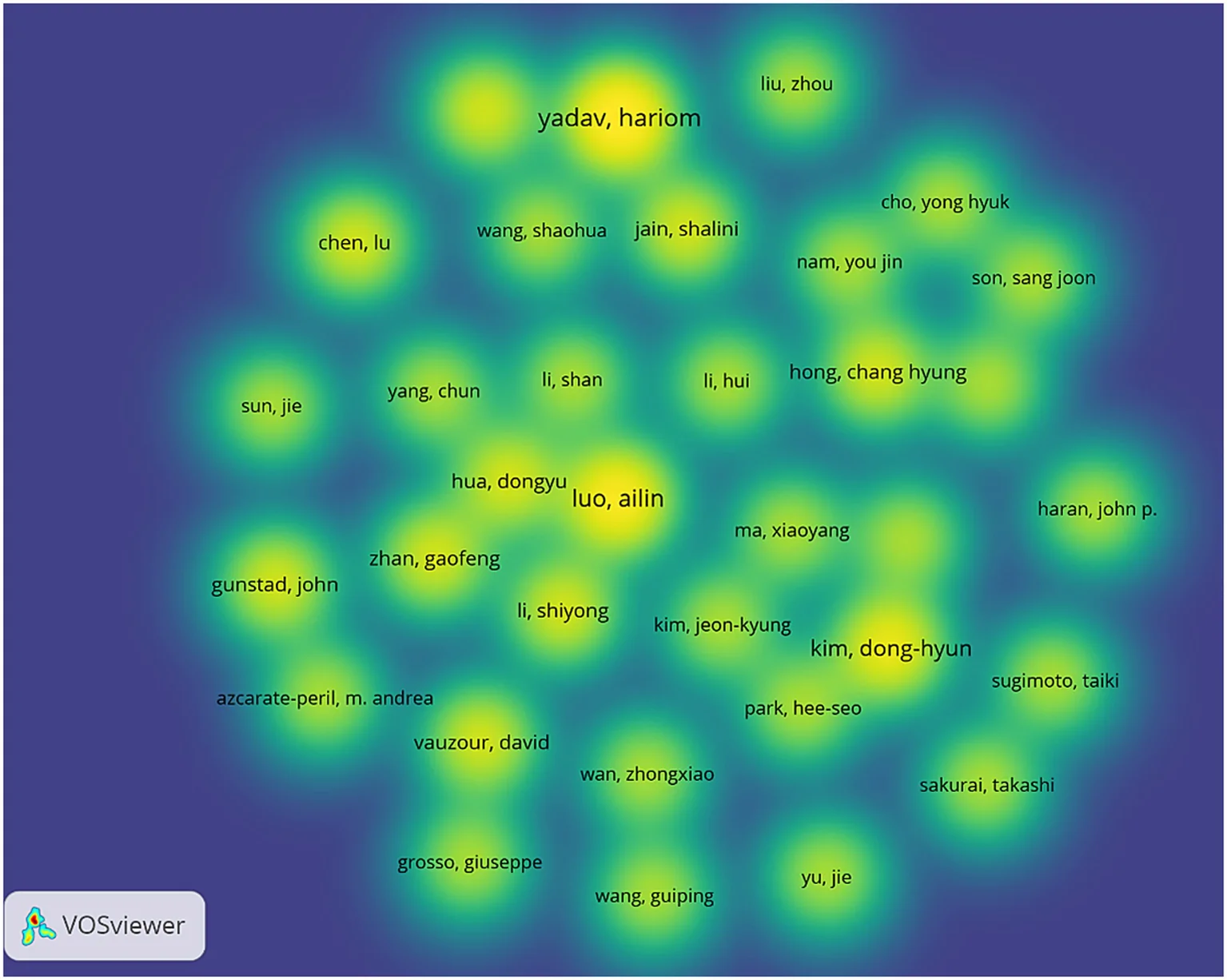
Author density map in the WOSCC database. The node size is related to the number of posts by the author, and the node color is related to the number of posts in recent years, the more posts the higher the color brightness.

**Table 4:** Top 10 authors sorted by total connection strength in the Scopus database.

Rank	Author	Documents	Total link strength
1	Hisada, T.	5	35
2	Murotani, K.	5	35
3	Niida, S.	5	35
4	Saji, N.	5	35
5	Sakurai, T.	5	35
6	Sugimoto, T.	5	35
7	Toba, K.	5	35
8	Tsuduki, T.	5	35
9	Wang, Z.	6	3
10	Zhang, Y.	10	3

**Figure 10:**
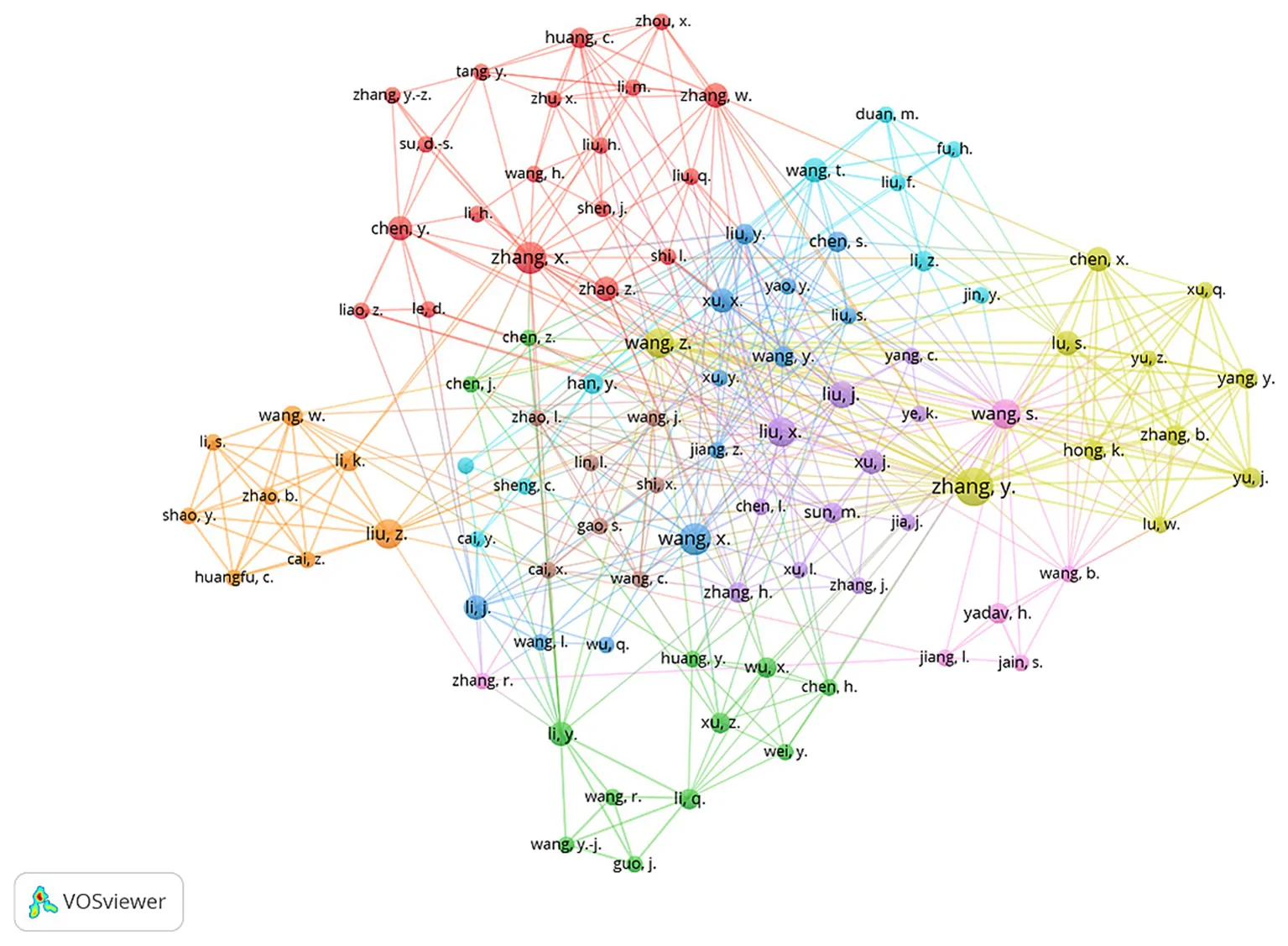
Cluster-based author collaboration map in the Scopus database. The diameter of the circle is positively correlated with the number of publications by the authors, while the density of the connecting lines intuitively reflects the intensity of the collaboration between the authors.

### Keyword analysis

Keyword co-occurrence analysis can visually and comprehensively reveal the research hotspots and trends in this field. In Figures 11, 12, in the WOSCC database, the co-occurrence mapping analysis of keywords shows that “gut microbiota” has the highest frequency of occurrence, followed by “Alzheimer’s disease,” “aging,” “probiotics,” “dementia” and “neuroinflammation.” Table 5 lists the top 10 keywords in terms of occurrence frequency. Additionally, in the Scopus database, in Figures 13, 14, the co-occurrence mapping analysis of keywords shows that “intestine flora “has the highest frequency of occurrence, followed by “aged “, “cognitive defect “, “cognition “, “gastrointestinal microbiome “and “Alzheimer disease “. Table 6 lists the top 10 keywords in terms of occurrence frequency. In terms of keyword frequency, Alzheimer’s disease emerged as the most frequently cited and intensively studied cognitive disorder within research exploring the relationship between gut microbiota and age-related cognitive impairment. This indicates that Alzheimer’s disease represents a significant focal point within the field of gut microbiota and cognitive dysfunction research. Inflammatory response, as a key pathological mechanism, has become a hot topic in mechanism research.

**Figure 11:**
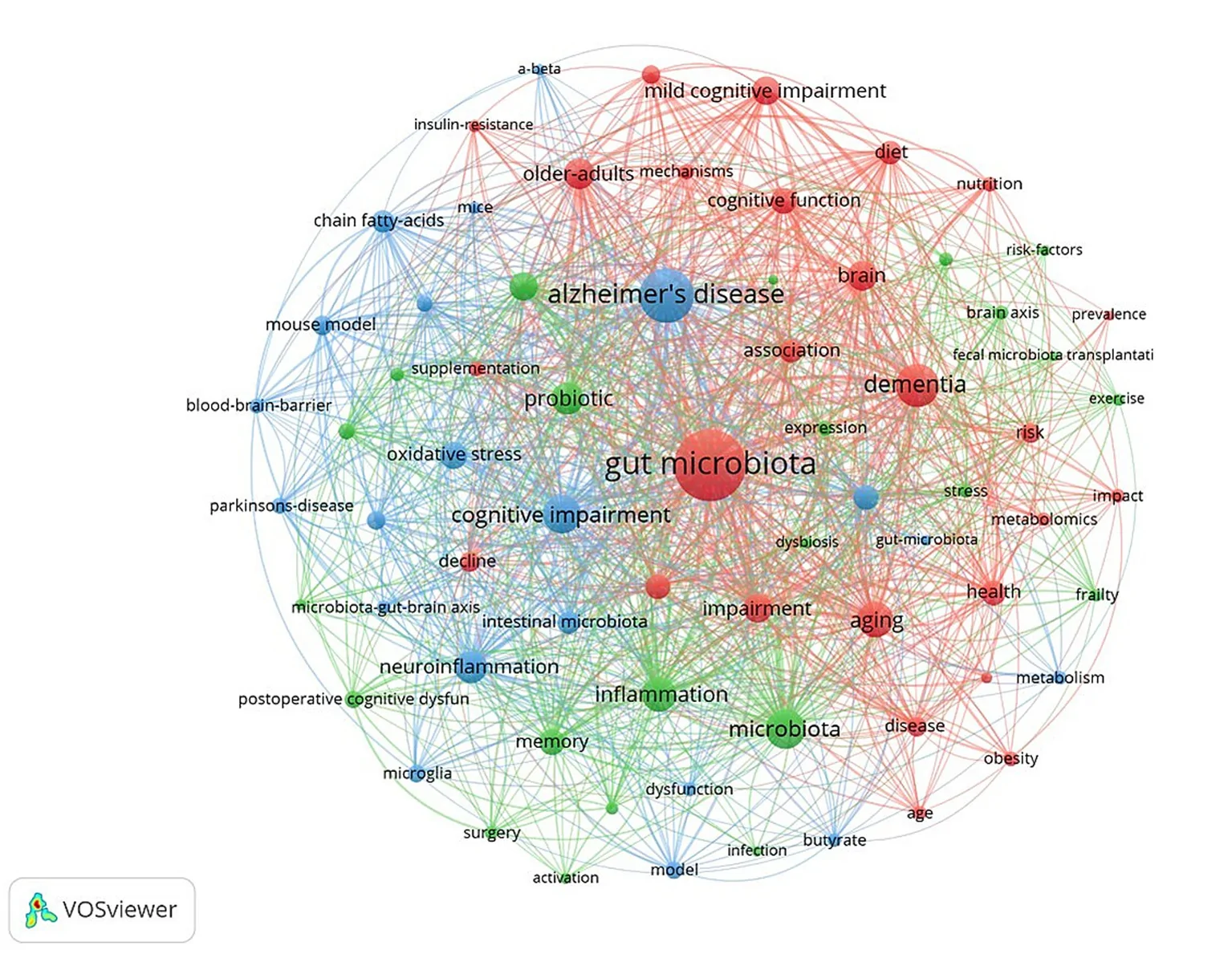
Cluster-based keywords collaboration map in the WOSCC database. The diameter of the circle is positively correlated with the frequency of the keywords, while the density of the connecting lines intuitively reflects the relevance of the keywords to each other.

**Figure 12:**
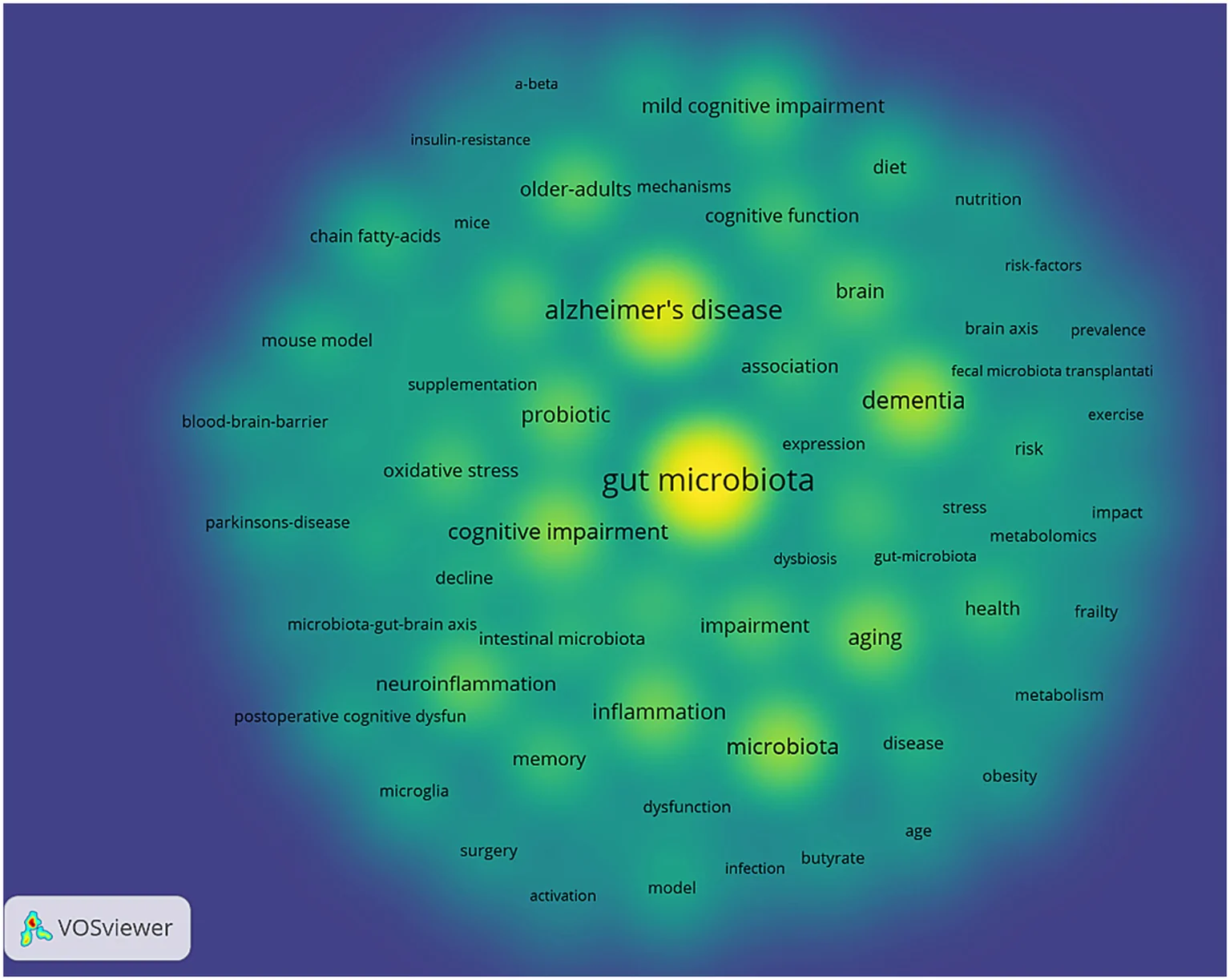
Key words density map in the WOSCC database. The node size is related to the number of posts by the key words, and the node color is related to the number of posts in recent years, the more posts the higher the color brightness.

**Table 5:** Top 10 keywords of word frequency in WOSCC database.

Rank	Keyword	Occurrences
1	Gut microbiota	234
2	Alzheimer’s disease	148
3	Dementia	99
4	Microbiota	88
5	Cognitive impairment	81
6	Aging	73
7	Inflammation	68
8	Probiotic	63
9	Neuroinflammation	61
10	Older-adults	58

**Figure 13:**
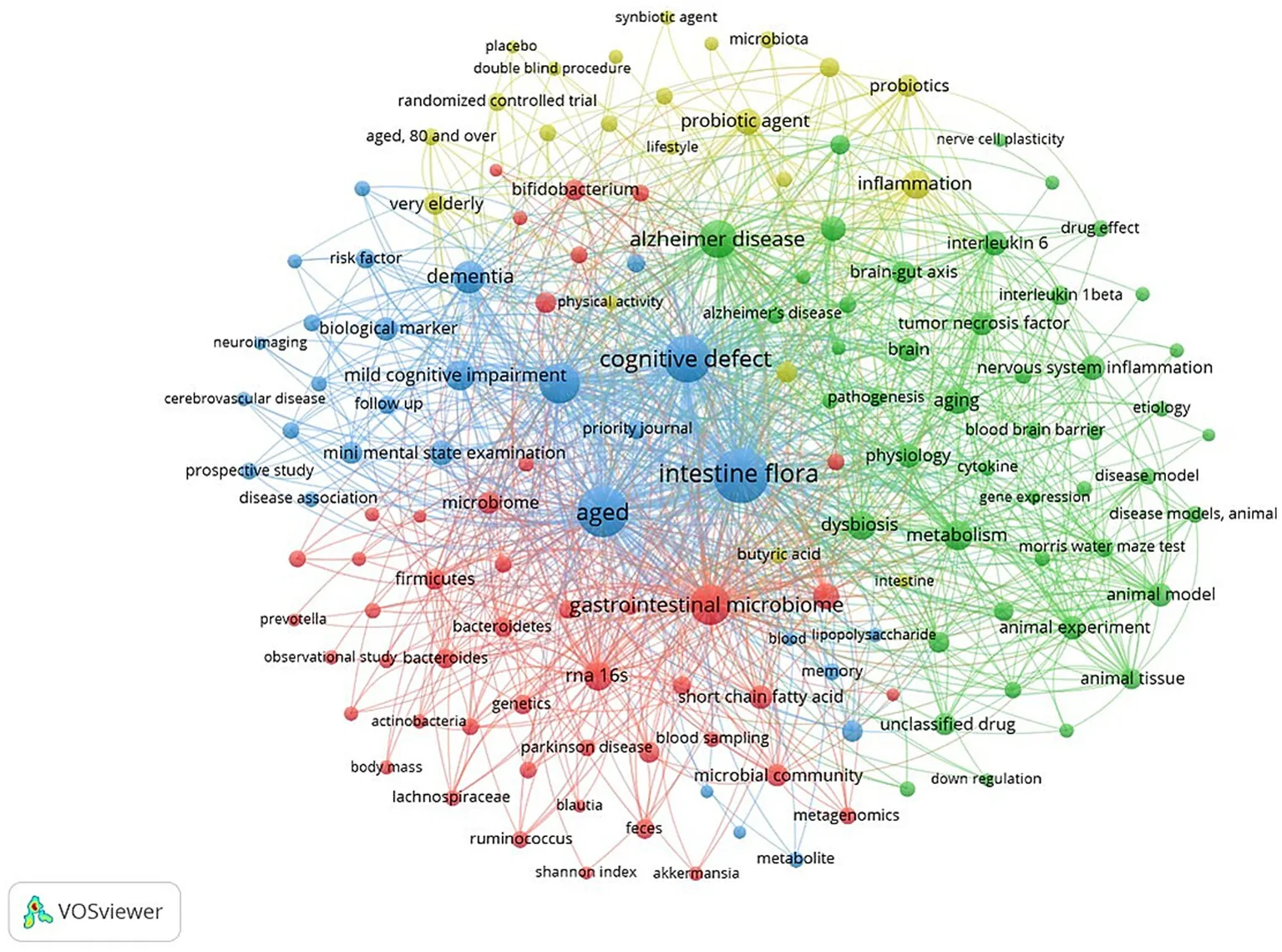
Cluster-based keywords collaboration map in the Scopus database. The diameter of the circle is positively correlated with the frequency of the keywords, while the density of the connecting lines intuitively reflects the relevance of the keywords to each other.

**Figure 14:**
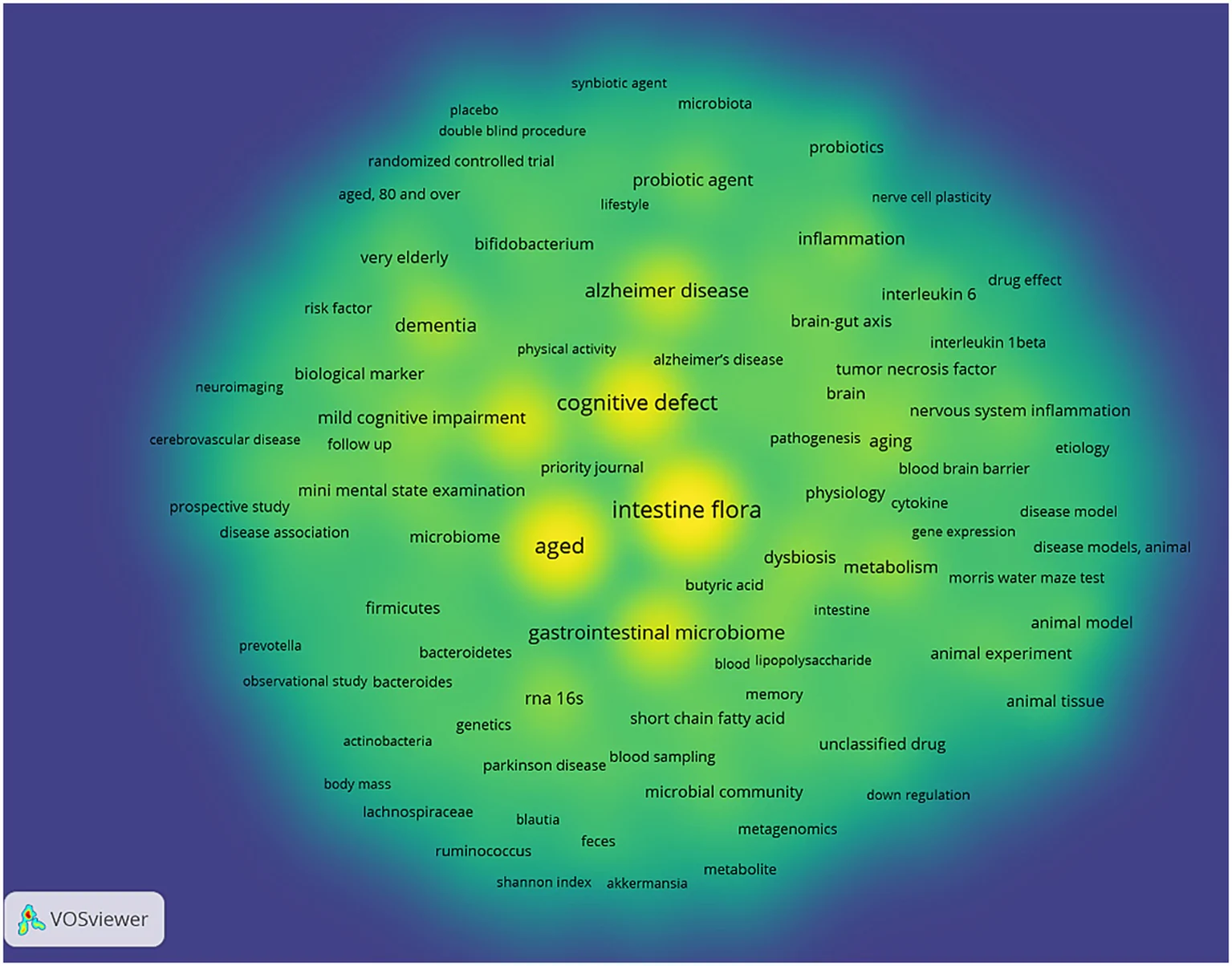
Key words density map in the Scopus database. The node size is related to the number of posts by the key words, and the node color is related to the number of posts in recent years, the more posts the higher the color brightness.

**Table 6:** Top 10 keywords of word frequency in Scopus database.

Rank	Keyword	Occurrences
1	Intestine flora	150
2	Aged	126
3	Cognitive defect	108
4	Cognition	84
5	Gastrointestinal microbiome	84
6	Alzheimer disease	74
7	Dementia	53
8	Metabolism	47
9	Mild cognitive impairment	45
10	RNA 16S	42

### Highly cited analysis

Highly cited literature often represents literature with high influence in the field. Highly cited and core literature focus on the latest research progress in the field. In the WOSCC database, Table 7 shows the top 10 most frequently cited documents. The article with the most cited literature is “Association of brain amyloidosis with pro-inflammatory gut bacterial taxa and peripheral inflammation markers in cognitively impaired elderly “published by cattaneo in the journal NEUROBIOLOGY OF AGING.

**Table 7:** The top 10 cited literatures in the WOSCC database.

Ranking	Citations	Author	Journal	Title
1	1,019	Cattaneo et al., 2017	Neurobiology of Aging	Association of brain amyloidosis with pro-inflammatory gut bacterial taxa and peripheral inflammation markers in cognitively impaired elderly
2	564	Kowalski and Mulak, 2019	Journal of Neurogastroenterology and Motility	Brain-Gut-Microbiota Axis in Alzheimer’s Disease
3	463	MahmoudianDehkordi et al., 2019	Alzheimers & Dementia	Altered bile acid profile associates with cognitive impairment in Alzheimer’s disease-An emerging role for gut microbiome
4	454	Nagpal et al., 2019	Ebiomedicine	Modified Mediterranean-ketogenic diet modulates gut microbiome and short-chain fatty acids in association with Alzheimer’s disease markers in subjects with mild cognitive impairment
5	371	Sun et al., 2019	Translational Psychiatry	Faecal microbiota transplantation alleviated Alzheimer’s disease-like pathogenesis in APP/PS1 transgenic mice
6	341	Kim et al., 2021	Journals of Gerontology Series	Probiotic Supplementation Improves Cognitive Function and Mood with Changes in Gut Microbiota in Community-Dwelling Older Adults: A Randomized, Double-Blind, Placebo-Controlled, Multicenter Trial
7	302	Haran et al., 2019	Mbio	Alzheimer’s Disease Microbiome Is Associated with Dysregulation of the Anti-Inflammatory P-Glycoprotein Pathway
8	297	Yang et al., 2020	Acta Pharmaceutica Sinica B	Probiotics modulate the microbiota-gut-brain axis and improve memory deficits in aged SAMP8 mice
9	232	Bertolotti et al., 2014	World Journal of Gastroenterology	Nonalcoholic fatty liver disease and aging: Epidemiology to management
10	231	Matt et al., 2018	Frontiers in Immunology	Butyrate and Dietary Soluble Fiber Improve Neuroinflammation Associated With Aging in Mice

### Comparison of findings between the WOSCC and Scopus databases

Although the Web of Science Core Collection and the Scopus database employ the same search period (1995–2025) and similar core concepts, their bibliometric results should be regarded as complementary rather than directly comparable. WOSCC included a total of 406 publications, while Scopus included 180. This discrepancy in numbers stems primarily from differences between the two databases in terms of coverage, indexing criteria and journal selection, rather than from any actual difference in research output. Consequently, metrics such as the number of publications, citations and average citations derived from the two databases are not statistically equivalent and should not be compared directly.

With regard to research hotspots, the two databases show a high degree of consistency. The most frequently occurring keywords in WoS were, in descending order, ‘gut microbiota’ (234 occurrences), ‘Alzheimer’s disease’ (148 occurrences), ‘dementia’ (99 occurrences), ‘microbiota’ (88 occurrences) and ‘cognitive impairment’ (81 occurrences); in Scopus, the most frequently occurring keywords were, in order, ‘intestinal flora’ (150 occurrences), ‘aged’ (126 occurrences), ‘cognitive defect’ (108 occurrences), ‘cognition’ (84 occurrences) and ‘gastrointestinal microbiome’ (84 occurrences). Although there are differences in specific terminology—partly due to Scopus’s greater reliance on automated indexing—both databases point to the same core themes: gut microbiota, aging, cognitive impairment/dementia and Alzheimer’s disease. This mutual corroboration enhances the reliability of the identified research hotspots.

At the author level, the most prolific author in WOSCCwas Hariom Yadav (7 papers), while the most prolific in Scopus was Y Zhang (10 papers). This discrepancy is not contradictory but reflects differences in the scope of the databases and in the disambiguation of author names. Consequently, the results of the author analyses from the two databases are presented separately and should be regarded as complementary descriptions of the same research community.

In summary, the analyses from WOSCC and Scopus corroborate one another on the main research themes, but their quantitative indicators are specific to their respective databases. The Scopus results serve as supplementary confirmation of the WoS findings, rather than as a basis for direct statistical comparison.

## Discussion

As population aging intensifies, cognitive impairment has emerged as a significant global public health challenge. Given its close association with the nervous system, research has primarily centred on the biochemical and molecular levels of aetiology. In recent years, scientific exploration has increasingly turned to the gut microbiome—a complex ecosystem comprising trillions of microorganisms—which is intrinsically linked to the host’s health and disease states. Emerging evidence indicates that the gut microbiota composition of individuals with cognitive impairment exhibits significant differences compared to healthy populations (Białecka-Dębek et al., 2021; Li et al., 2018; Li et al., 2021). This study, based on bibliometric methods, utilized VOSviewer software to construct a scientific knowledge graph to conduct a visual analysis of the research progress of gut microbiota in the field of cognitive impairment in the elderly. This study systematically analyzed the research hotspots in this field from aspects such as publication trends, countries/regions, institutions, journals, authors, and keywords.

### Current status of research

This study examined 406 included papers after filtering. Research on the connection between gut microbiota and cognitive impairment has been active, as seen by the consistent yearly rise in publications. The scientific community’s awareness of this field’s potential worth and the growing research activity in it are both reflected in this growth tendency. Development in this subject has been fueled by technological advancements, increased societal demand, and coordinated efforts by governments and academic institutions—through research funding, worldwide cooperative initiatives, and policy support. Among these, Nanjing Medical University and Zhejiang University are tied for first place in terms of influence. Yadav, Hariom had the highest publication count in the WoS database, although this metric should be interpreted in light of the team-based nature of research in this field. Rankings of high-output authors based on publication volume have inherent methodological limitations. Several authors from the same laboratory or research group may appear on the list simultaneously due to co-authorship of the same set of papers, thereby exaggerating the team’s overall representation; conversely, independent researchers may be excluded despite having published a small number of pioneering papers, simply because their total publication output is low. Consequently, the author rankings in this study should be understood as a reflection of ‘publication activity’, rather than an assessment of individual academic influence, contribution or innovation. The majority of current study in this field is still focused on North America and Europe. Western countries have high-quality research institutions and actively participate in worldwide scientific networks and cooperative initiatives, as evidenced by their average citation count of over 48. This strengthens their role as essential hubs within the global research ecosystem by promoting data sharing and resource integration. Because of the substantial rise in research funding in recent years, which has given studies on gut microbiota and cognitive impairment strong support, China leads the world in the amount of literature produced in this topic. Second, China offers special benefits for carrying out extensive cohort research and clinical trials due to its massive population base and plentiful clinical resources. Additionally, Chinese research institutes are expanding their partnerships with top international research groups, which will increase their scholarly impact in this area.

According to current trends, strengthening collaboration between research institutions through the creation of interdisciplinary teams—especially those that span several nations and regions—will make it easier to integrate knowledge from other domains. This strategy will make it possible to investigate the complex connection between gut microbiota and cognitive impairment in greater detail. Research on cognitive impairment and the gut microbiome is greatly advanced by highly referenced journals and publications. They emphasize the translation and application of discoveries, going beyond basic research. These articles will continue to direct research priorities and cutting-edge trends in the area as technology develops and research expands.

### Research hotspots and trends

The research hotspots and trends in this sector can be graphically and thoroughly shown through keyword analysis, which can also provide guidance for future research (Li et al., 2022). The comprehensive analysis of key words and co-citations of literature shows that the current research on gut microbiota in the field of cognitive impairment in the elderly mainly focuses on the following directions: different cognitive impairment diseases in elderly patients, among which AD is the core research object; at the level of mechanism pathways, inflammation, as a key pathological mechanism, has long been of concern. The gut-brain axis has been identified as an important signaling pathway by which the gut microbiota affects cognitive function. In addition, probiotics for diet management have become the latest research hotspot in the field of intestinal flora management. Figure 15 shows the research hotspot change.

**Figure 15:**
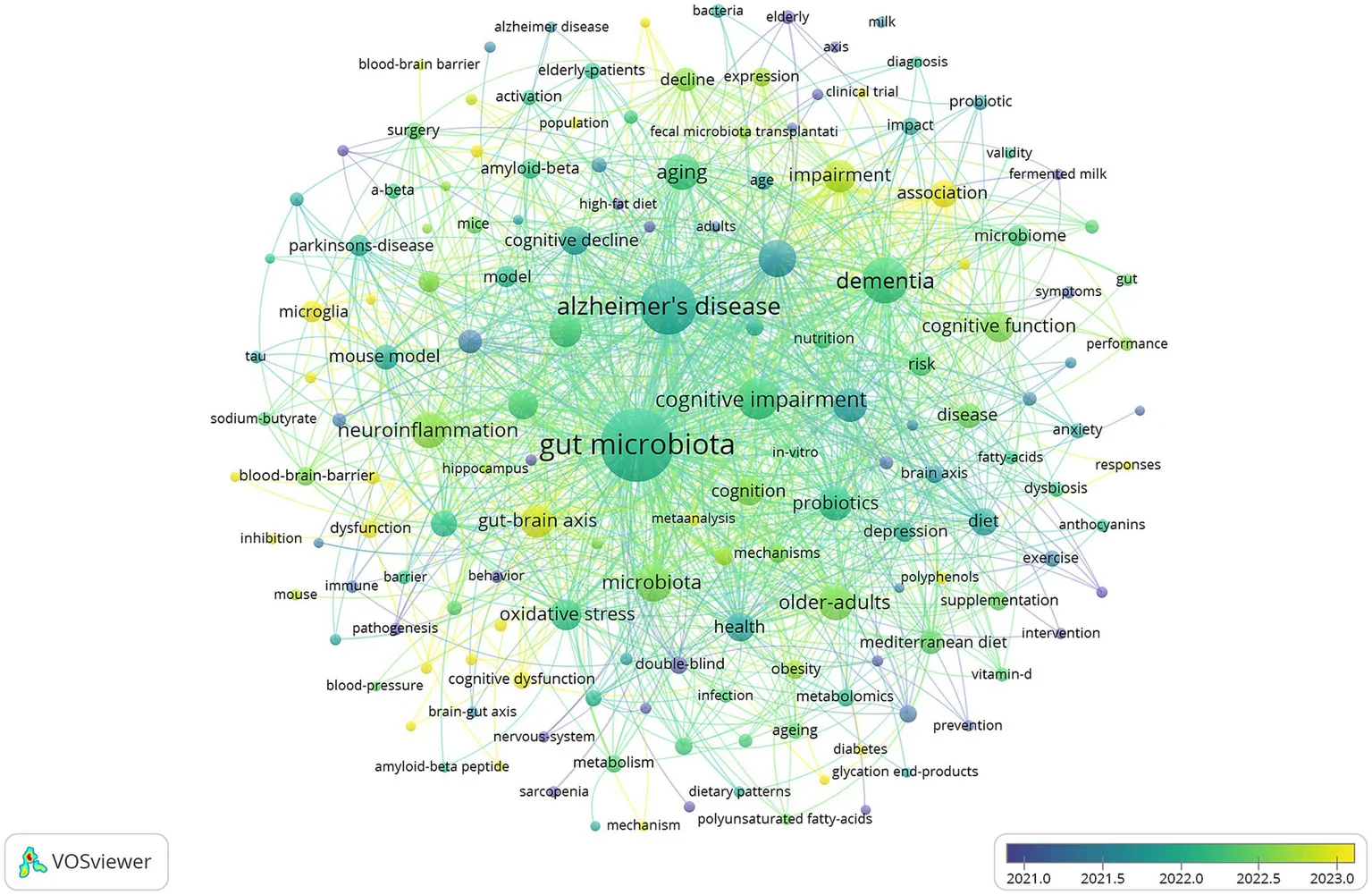
Changes in research hotspots within the WOS database. The node size is related to the popularity of posts containing that keyword, and the node color is related to the publication date of posts in recent years; the more recent the publication date, the higher the color brightness.

Research in this area was still at the “exploratory” stage before 2016. Nonetheless, some research has started to identify possible links between the gut microbiota and age-related cognitive decline, setting the stage for more studies (Pérez Martínez et al., 2014). Significant differences exist in the gut microbiota characteristics of elderly populations with varying cognitive states, such as vascular dementia and depression (Yin et al., 2015; Simpson et al., 2021; Liu L. et al., 2023). Among these, Alzheimer’s disease patients exhibit higher abundance of Bacteroides and Proteus in their gut microbiota, while Firmicutes and Bifidobacteria are relatively less abundant (Chandra et al., 2023). The depth and scope of study at the time were hampered by the scarcity of multi-omics analytic tools and high-throughput sequencing technologies. Research in this area has been expanding quickly since 2017. The emergence and broad acceptance of the “microbiome-gut-brain axis” theory, advances in high-throughput sequencing, metabolomics, and neuroimaging techniques, the impetus from high-impact studies, and the interdisciplinary convergence of microbiology, neuroscience, immunology, and psychology have all contributed to this expansion. The processes by which gut microbes affect cognitive impairment and microbiome-based intervention techniques are becoming more and more the focus of current research (Chen et al., 2021; Silva et al., 2020). This shift indicates that research has progressed from preliminary exploratory studies to in-depth investigations into the role of the gut microbiota in the pathogenesis of depression. The role of gut microbiota and their metabolites in cognitive impairment exhibits a double-edged sword characteristic: while contributing to disease pathogenesis (Medzhitov, 2007; Jakobsson et al., 2010; Rieder et al., 2017), they also offer novel therapeutic avenues (Wang, 2025; Sharon et al., 2016; Kim and Shin, 2019; Bravo et al., 2011).

### The development directions of future research

Fundamental research is the main emphasis of the current literature in this topic. Even though there has been a lot of progress, there are still many issues that need to be resolved. For example, more research is needed to confirm the safety and effectiveness of microbiome-based treatments such faecal microbiota transplantation in clinical settings. Probiotics and faecal microbiota transplantation are two examples of current therapies that are primarily based on small-scale pilot trials with uneven effects and no evidence of long-term follow-up. The design of meticulously carried out randomized controlled trials to investigate tailored intervention approaches should be the focus of future initiatives. These could involve examinations of their specific cognitive advantages and safety profiles, as well as diet or microbiota manipulation techniques customized to individual microbial profiles. Additionally, the potential of gut microbiota as early indicators should be actively investigated, and non-invasive, sensitive instruments for early warning and staging of cognitive impairment should be developed. The interactions between host and environmental factors have not received enough attention in previous studies.

Increasing the depth of population-specific research: At the moment, the majority of studies focus on populations in Europe and America, whereas there is comparatively little study data on people in East Asia. Larger-scale population-specific research is still required, although teams from China and South Korea have already started to close this gap. Additionally, a more thorough gut-brain axis regulatory network is created by combining metagenomics, metabolomics, proteomics, and neuroimaging data. This creates new targets for accurate nutritional intervention and early diagnosis, such as using biomarkers in cerebrospinal fluid (CSF), blood test indicators, detailed brain scans, artificial intelligence algorithms, etc. (Thakur et al., 2025). It is crucial to conduct further aging trials, better integrate multi-omics data, and focus more on brain-gut axis research.

### Strengths and limitations

In order to provide a preliminary understanding of the knowledge structure and evolutionary patterns in the field of gut microbiota and age-related cognitive impairment, this study uses comprehensive bibliometric analysis. Its methodological rigor and visual presentation are its strongest points. Specifically, complementary searches were conducted across both the Web of Science Core Collection and Scopus databases. The fundamental corpus’s comprehensiveness and the depth of its analytical aspects were significantly ensured by the extensive mapping that was done across nations, institutions, authors, keywords, and citation networks. This offers a mathematical foundation for understanding this field’s macro-level environment.

However, there are a number of limitations to this study that should be taken into account when interpreting the results. First, the literature search was limited to English-language research articles and reviews at the corpus level, and the retrieval approach depended on certain keywords and Boolean logic, which may have introduced bias or resulted in the deletion of pertinent material. Second, this study did not conduct sensitivity tests on the search strings, manual verification or supplementary searches in PubMed/MEDLINE or Embase; consequently, the results may be subject to selection bias. Furthermore, a high threshold may indeed result in low-frequency but important emerging themes being excluded. What is more in terms of methodology and reproducibility, the precise replication of analytical results may be impacted by the lack of clarity in the particular parameter settings (such clustering resolution) used in the VOSviewer software during analysis. The methodology’s interpretive limitations are the most significant drawback. Bibliometrics is essentially a descriptive and mapping tool that can successfully disclose knowledge structures, collaborative networks, and research trends. It cannot, however, evaluate the actual clinical effectiveness of different therapeutic strategies or directly clarify the precise biological processes by which gut microbes affect cognition. Therefore, further experimental research and clinical evidence are needed to carefully validate the discussion in this study addressing future mechanistic exploration and translational therapies, which is extrapolated from the metrological findings.

## Conclusion

This study employs bibliometric analysis software such as VOSviewer to conduct a systematic visual analysis of literature linking cognitive impairment with the gut microbiota. By comprehensively examining the current research landscape, key areas of focus, and emerging challenges within this field, it aims to provide insights and reference value for future investigations. Research into cognitive impairment and the gut microbiota is advancing rapidly, yet numerous challenges remain. Future research paradigms will inevitably evolve toward systematic, multidimensional, and deep multi-omics integration. This emerging trend centres on transcending singular microbiome sequencing to synchronously integrate metagenomic, metabolomic, proteomic, and host transcriptomic/epigenomic data, while establishing longitudinal associations with neuroimaging and refined clinical phenotypes. Through such integration, research will not only map static ‘microbe-host’ association landscapes but also dynamically elucidate the specific molecular pathways and causal networks through which gut microbiota influence cognitive function via computational modelling. This will transform current associative evidence into mechanistic models with clear biological interpretations. Ultimately, this trend will propel the field beyond mere phenomenological description toward a new era of early warning, precise phenotyping, and targeted interventions based on multi-omics biomarkers. This approach will deliver genuinely translatable, personalised solutions for the prevention and treatment of age-related cognitive impairment.

## Data Availability

The raw data supporting the conclusions of this article will be made available by the authors, without undue reservation.
